# The high burden of infant deaths in rural Burkina Faso: a prospective community-based cohort study

**DOI:** 10.1186/1471-2458-12-739

**Published:** 2012-09-05

**Authors:** Abdoulaye Hama Diallo, Nicolas Meda, Halvor Sommerfelt, Germain S Traore, Simon Cousens, Thorkild Tylleskar

**Affiliations:** 1 Centre MURAZ Research Institute, Ministry of Health/Burkina Faso, PO Box 390, Bobo-Dioulasso, Burkina Faso; 2 Centre for International Health (CIH), University of Bergen, PO Box 7804, N-5020, Bergen, Norway; 3 Division of Infectious Diseases Control, Norwegian Institute of Public Health, Oslo, Norway; 4 Regional Nursing School of Public Health (ENSP/Bobo), Bobo-Dioulasso, Burkina Faso; 5 London School of Hygiene and Tropical Medicine, Department of Infectious Disease Epidemiology (IDEU), London, UK

**Keywords:** Infant mortality, Risk factors, Rural areas, Burkina Faso

## Abstract

**Background:**

Infant mortality rates (IMR) remain high in many sub-Saharan African countries, especially in rural settings where access to health services may be limited. Studies in such communities can provide relevant data on the burden of and risk factors for infant death. We measured IMR and explored risk factors for infant death in a cohort of children born in Banfora Health District, a rural area in South-West Burkina Faso.

**Methods:**

A prospective community-based cohort study was nested within the PROMISE-EBF trial (NCT00397150) in 24 villages of the study area. Maternal and infant baseline characteristics were collected at recruitment and after birth, respectively. Home visits were conducted at weeks 3, 6, 12, 24 and 52 after birth. Descriptive statistics were calculated using robust standard errors to account for cluster sampling. Cox multivariable regression was used to investigate potential risk factors for infant death.

**Results:**

Among the 866 live born children included in the study there were 98 infant deaths, yielding an IMR of 113 per 1000 live births (95% CI: 89–143). Over 75% of infant deaths had occurred by 6 months of age and the post neonatal infant mortality rate was 67 per 1000 live births (95% CI: 51–88). Infections (35%) and preterm births complications (23%) were the most common probable causes of death by 6 months. Multivariable analyses identified maternal history of child death, polygyny, twin births and poor anthropometric z-scores at week-3 as factors associated with increased risk of infant death.

**Conclusions:**

We observed a very high IMR in a rural area of Burkina Faso, a country where 75% of the population lives in rural settings. Community-based health interventions targeting mothers and children at high risk are urgently needed to reduce the high burden of infant deaths in these areas.

## Background

Millennium Development Goal 4 (MDG-4) calls for a two-thirds reduction in the under-five year mortality rates (U5MR) between 1990 and 2015 [1]. An important contributor to under-five mortality is infant mortality, which in 2010 represented 70% of all child deaths, i.e. 5.4 million deaths of an estimated global burden of approximately 7.7 million child deaths under-five years of age [2]. An important component of infant deaths is neonatal deaths.

The worldwide infant mortality rate (IMR) was estimated by the United Nations Children Fund (UNICEF) to be 42 per 1000 live births in 2009, with Sub-Saharan Africa the region with the highest rate (81 per 1000 live births) [3]. However, recent publications have suggested a substantial reduction in post-neonatal mortality rates over the last twenty years (1990–2009) with an annual average reduction globally of 2.2% [2,4-6]. But, the rate of IMR reduction in Sub-Saharan Africa has been lower than in Asia [3,7]. In addition, there are substantial regional and within-country variations in IMRs and their reduction over time [2,3,6]. Within Africa, Central and West Africa are the two regions with highest IMRs, as high as 92 per 1000 live births, 10 points above the Sub-Saharan Africa average [8].

An important observation on recently published estimates on child deaths including infant mortality is the shifting figures of the global estimates of child mortality, owing partly to the lack of reliable statistics in many countries where the burden is the highest, and also to changes in statistical methods used for the estimates [2-7]. Several reports have called for the collection of high quality data in low-income countries, especially in rural areas of Africa where the decrease in child mortality has been slow [4-6,9,10].

With just a few years to go until the MDG-4 deadline, several reports suggest that very few Sub-Saharan African countries are likely to meet this goal by 2015 [4,6]. It is therefore important to monitor the MDG-4 progress in the countries with the highest IMRs in order to provide more reliable statistics of this outcome and also to guide policy makers for relevant and contextualized local health programs.

Monitoring child mortality in a country like Burkina Faso (West Africa) where over 75% of the population lives in rural settings [11] will benefit from studies that are conducted in rural communities, also those with poor access to health care facilities, and when possible using a prospective study design.

The present study seeks to contribute to this global effort of quality data production and reports on the findings from a community-based cohort study that sought to measure IMR in a rural area in Burkina Faso and explore possible risk factors for infant death.

## Methods

### Study area

**Figure 1:**
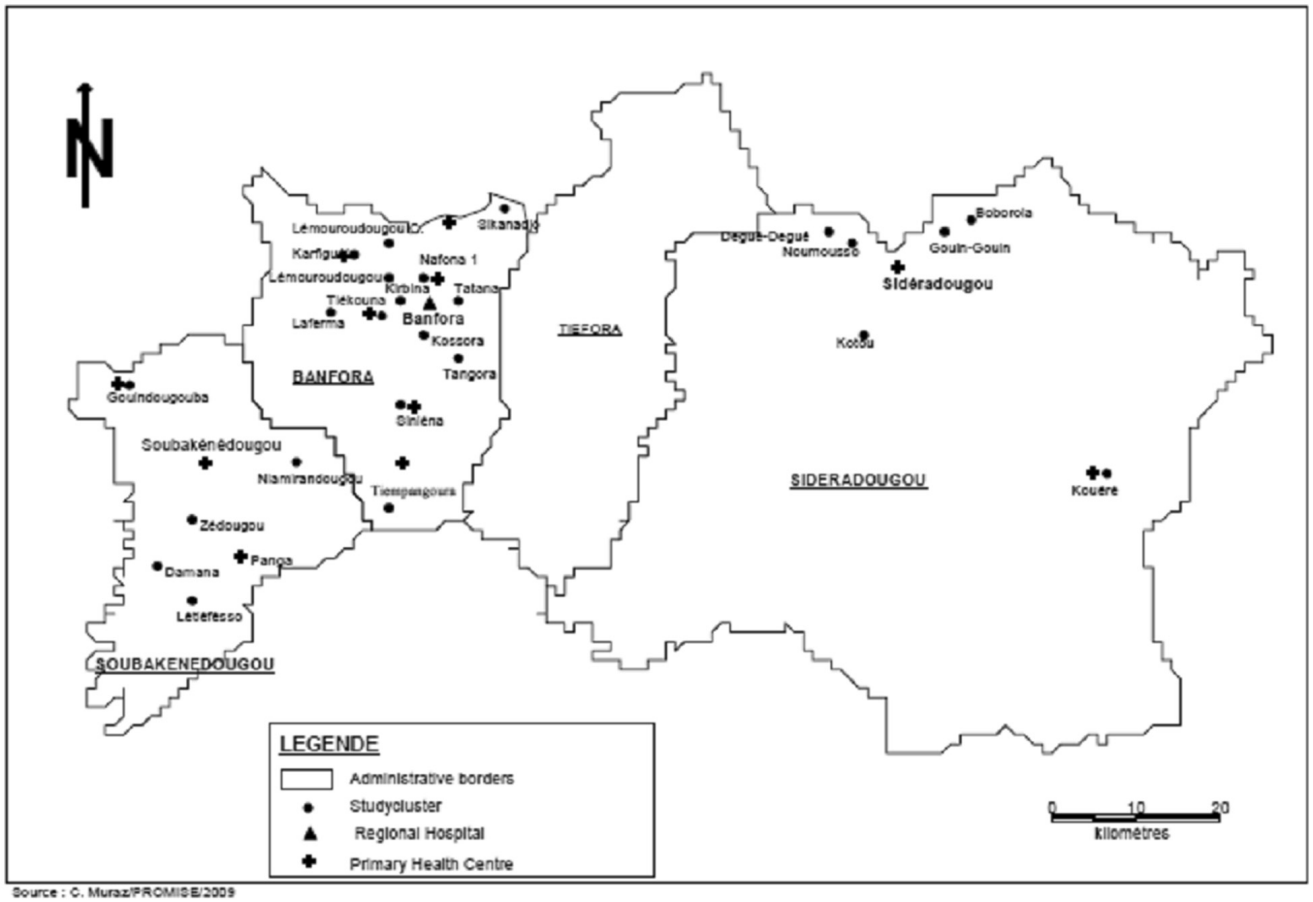
Overview of the study area.

The study took place in Banfora Health District, in the south-west of Burkina Faso close to the border with Cote d“Ivoire. The district covers an area of 6,300 km^2,^ and had an estimated population of 282,000 in 2010 with three major ethnic groups, the Gouin, the Karoboro and the Dioula [11]. The area experiences an annual rainfall of 950 to 1250 mm during a 6-month rainy season (May-Oct). Farming and animal husbandry are the main activities in the rural areas while the town of Banfora with a population of 75,000 is a flourishing trading centre [11]. The study was conducted in three subcounties, Banfora, Soubakénédougou and Sidéradougou (Figure 1).

In 2006, the district health system consisted of 60 primary health facilities and one regional hospital in the town of Banfora. The ratios of health personnel to population in 2010 were estimated to be approximately 1:5300 for nurses, 1:5200 for midwives, and 1:40000 for physicians [12].

Based on official reports for the study area from the Ministry of Health in Burkina Faso, over 94% of pregnant women attended antenatal care (ANC), 77% were reported to deliver in a health facility and 66% of children aged 12–23 months were reported to have received the full set of EPI vaccines in 2010 [13]. HIV-prevalence is low in the rural areas of Banfora region and was estimated to be of 0.6% among the 15–49 years-old in 2010 [13]. The 2006-national census in Burkina Faso reported Banfora to have an IMR of 101 and a U5MR of 165 per 1000 live births [11].

### Study design

A cohort study was nested within the PROMISE-EBF trial ( http://www.clinicaltrials.govNCT00397150 ), a community-based, cluster-randomized trial to promote exclusive breastfeeding (EBF) through individual peer-counselling, which was implemented in 24 villages of Banfora Health District as reported elsewhere [14,15]. Children born to all pregnant women enrolled in both arms of the main PROMISE-EBF trial formed a prospective cohort that was followed until 12 months of age.

### Sample size

The PROMISE-EBF trial sample size was calculated using prevalence of EBF and diarrhea at 12 weeks of child age as primary outcomes [14,15]. No sample size estimation was done for infant deaths. However, post-hoc analyses showed that the sample of 866 newborns enrolled would enable us to estimate the IMR with a precision of  ± 2% based on estimates from the 2006-national census in Burkina Faso [11] and a confidence level of 95%.

### Recruitment and follow-up of study participants

The details of participants’ enrolment and follow-up for the first 6 months are reported elsewhere [14,16]. In summary, pregnant women were identified in each study village by female “recruiters” over a one year period (June 2006 to May 2007) through weekly household visits. In 23 villages with a mean population of 1330, a random sample of up to 4 pregnant women per village was selected monthly. In the 24^th^ village (Siniéna) with a population of nearly 5000, we sampled 8 women per month instead of 4. Women were recruited into the EBF-trial if they met the study inclusion criteria which were as follows: pregnancy of 7 months or more, intention to remain in the village for the next 12 months, plan to breastfeed the child, absence of any severe maternal disease or mental handicap which could prevent either breastfeeding or cooperation and provision of individually written and informed consent.

While the main EBF-trial included only singleton live births and planned a follow-up for 6 months, we report here on all live born children of enrolled mothers, including those who had multiple births. Children were followed-up by trained data collectors, irrespective of trial arm until they were 12 months or older. Data collection visits were scheduled at recruitment and after birth at day 7 and at weeks 3, 6, 12, 24 (± 7 days for each visit) and at 12 months. Mothers who were not at home for a scheduled home visit were revisited by data collectors three times before the visit was considered as missed. Data collection lasted from March 2006 to November 2008.

Maternal baseline data (age, parity, medical history, household assets, etc.) were recorded at enrolment. Pregnancy outcomes and newborn baseline data were collected during the day-7 visit or at the earliest completed visit after birth. Newborn birth weight was recorded from the child health card when available. From week-3, we recorded information on the child’s feeding pattern and growth. Deaths at any time after birth were recorded. Infant weight and height were measured at each home visit using Seca®872 scales and a Seca®210 infantometer ( http://www.seca.com), respectively. Weight was recorded to the nearest 0.10 kg and height was measured to the nearest 0.5 cm. All interviews were conducted in the mother’s local language to improve comprehension and cooperation.

A standard verbal autopsy (VA) questionnaire [17] was used to capture information on the circumstances surrounding infant deaths and was filled within 4–6 weeks. However, narrative items describing the causes of death were completed only for infants who died before 6 months of age and so cause of death was only assessed for deaths before 6 months of age. Two independent physicians reviewed the VAs to assign probable causes of death using a hierarchical grouping adapted from the Child Health Epidemiology Reference Group Classification [18] and ICD-10. Deaths during the neonatal period were classified into the following sequential cause groups: congenital defects, tetanus, trauma/surgical, preterm birth complications, birth asphyxia, sepsis/pneumonia, diarrhoea/gastroenteritis, other/unknown. Postneonatal deaths were classified into the following causes: diarrhoea/gastroenteritis, pertussis, measles, injury/surgical, meningitis, pneumonia/acute respiratory tract infection, malaria, malnutrition, other/unknown. Multiple causes were allowed, although only the primary cause of death is reported here. The opinion of a senior paediatrician was sought in cases of disagreement between the two physicians.

Data collection was done using handheld computers (PDAs) with the Epihandy software ( http://www.openXdata.org) for visits up to 6 months, and with paper forms for the 12 month visit.

### Outcomes and exposures definitions

We used the WHO’s standard definitions of neonatal (i.e. death of a live born newborn within 28 days), post-neonatal (i.e. death of an infant between 1–12 months) and infant death (i.e. the death of any live born infant before 12 months of age). The main exposures included in analyses were maternal baseline characteristics (age, parity, education, socioeconomic status, use of health services and medical history) and newborn characteristics (season of birth, sex, twinship and anthropometry). Children with birth weight <2500 g were considered as low birth weight. Anthropometric status was assessed using WHO’s standards ( http://www.who.int/childgrowth/en/). Children were classified as wasted, stunted or underweight if their relevant z-score was below −2. A child with any z-score < −2 at 3 weeks of age was defined as having a “low anthropometric score”.

### Data entry and analysis

Data collected on paper questionnaires were entered by two independent clerks using Epidata 3.1 ( http://www.epidata.dk), cleaned and merged with the cleaned datasets from the electronic questionnaires. Data were analyzed with STATA/SE 11.0 (Statacorp, College Station, Texas).

Summary statistics of continuous and discrete variables of mothers and infants were produced. The 95% confidence intervals (CI) of proportions were calculated using robust standard errors to account for the cluster sampling of the PROMISE-EBF trial.

Risk of death by one year of age (commonly known as IMR) was calculated as the proportion of infant deaths per 1000 live births and a 95% CI calculated using a robust standard error.

Mortality rates were estimated using survival analysis and are reported per 1000 person-years of observation (PYO). A Kaplan-Meier plot was produced to show cumulative risk of death until 12 months. Between-cluster variation in mortality rates was assessed using a likelihood ratio test (LR test) with a random-effects Cox regression model.

Potential risk factors for infant deaths were screened through univariable Cox regression models for three age ranges (0–6 months, 1–12 months and 0–12 months). These analyses took account of possible clustering (fitting Cox Gamma shared frailty models in STATA/SE 11.0) and only variables with a p <0.25 in Wald-statistic tests were retained for further exploration. We explored interactions between polygyny and several maternal baseline variables including distance to nearest health facility, education, parity, ANC visits, and health facility delivery. We also looked at interactions between health facility delivery and maternal education or parity. Based on Mosley and Chen’s model [19] for risk factors assessment in child mortality, we conducted multivariable Cox regression models adjusting for distance to nearest health facility, maternal history of child death, newborn’s season of birth and sex considered as potential confounders. Covariates that remained associated with infant death risk (p < 0.05) in the adjusted models and that met major criteria for causal inference [20] were considered as risk factors.

### Ethical and administrative clearances

The study was approved by the Institutional Review Board of Centre MURAZ in Burkina Faso (N°013/2005/CE-CM) and by the Western Regional Committee for Medical and Health Research Ethics in Norway (Sak No05/8197). Administrative clearances were sought from the national and regional health authorities of Burkina Faso. All study participants were requested to provide individually written and informed consent prior to enrolment. All mothers and infants included in the study were offered free care and medication in local health facilities for the duration of the study, for illnesses related to lactational problems (mastitis, breast abscess) and infections (pneumonia, diarrhoea and malaria).

## Results

### Baseline data

**Figure 2:**
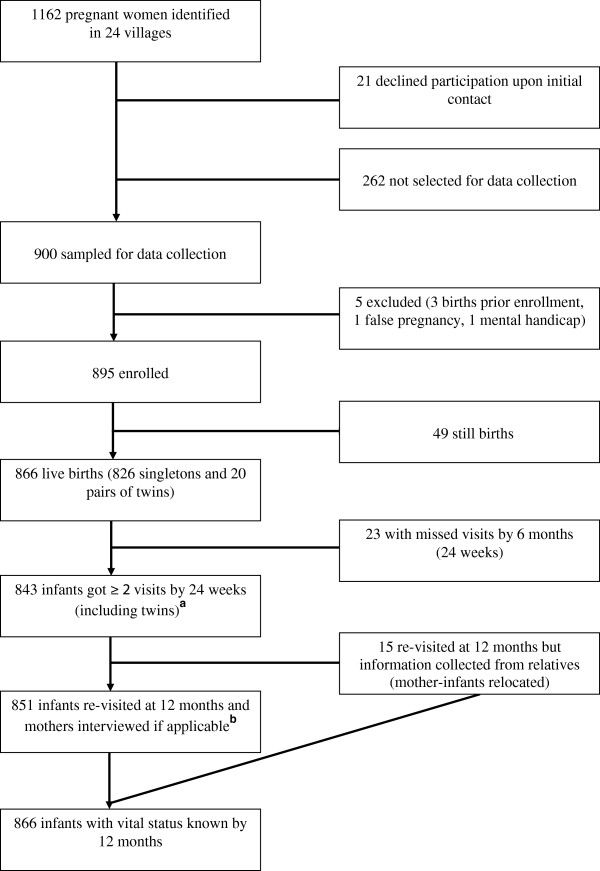
**Flow chart of the cohort study in Banfora Health District, Burkina Faso.**^a^The initial EBF-trial focused only on singleton births and was scheduled at weeks 3, 6, 12 and 24. The cohort follow-up included twins and added further visits at day 7 and 12 months after birth. ^**b**^Mothers whose children had died and were initially interviewed (verbal autopsy) were visited for formal greetings but not re-interviewed.

Over a one-year period, 1162 pregnant women were identified, 895 of whom were enrolled in the study (Figure 2). There were a total of 866 live births including 20 pairs of twins. The vital status of all infants was known at 12 months of age, although for 15 children (1.7%), data were collected from a close relative as they had relocated out of the study clusters.

There was an average of 36 live births recruited per village, with a range of 16–86. One village, Siniéna had a population of 5,000 and a higher number of births recruited (N = 86). Only six villages (25%) had a local health facility and 31% of infants were born in these villages. The proportion of twins births was particularly high (22%) in one village (Nafona1).

The average maternal age at enrolment was 26.4 years (SD = 6.5) and the median parity 3 (IQR = 2-4). High proportions of mothers were multiparae (84%) and had no formal education (80%). Almost half (48%) lived in polygynous households. About 60% of multiparous women had experienced a previous child death. Only 18% of the mothers had attended ≥ 2 antenatal care visits (ANC) by the time of recruitment and the proportion of health-facility deliveries was only 38%. The number of ANC-visits and the proportion of facility-deliveries varied inversely with parity (chi^2^ tests for trend, p < 0.001 in both cases). We found no evidence that maternal education or an index of household assets was associated with the use of health services during pregnancy (p = 0.41 and p = 0.48, respectively) or at delivery (p = 0.16 and p = 0.42, respectively).

Only 295 newborns, all born in health facilities, had a birth weight recorded on the child health card. Among the 723 children with weight and length measured at week-3, all three median z-scores were negative (Table 1), indicating poor anthropometric status among infants of this population. Overall, 212 infants (29%) at week-3 visit had a low z-score (<−2 SD) of whom 21 (3%) were stunted, 71 (10%) were wasted, 25 (3%) were underweight, and 95 (13%) had two or three of these outcomes together.

**Table 1:** Maternal and infant baseline, number of infant deaths and infant mortality rates in a cohort of 866 live births in Banfora Health District, Burkina Faso

	**Mothers (n = 846)**	**Live births (n = 866)**	**Infant deaths**	**Infant mortality rates (per 1000 live births)**
	***N (%)***	***N (%)***	***(N = 98)***	
Categorical variables				
Area of residence				
Banfora	418 (49)	429 (50)	56	130.5
Soubakénédougou	208 (25)	212 (24)	18	85.0
Sidéradougou	220 (26)	225 (26)	24	106.7
Distance to nearest health facility				
≤5 km	406 (48)	419 (48)	55	131.3
>5 km	440 (52)	447 (52)	43	96.2
Maternal age				
<20	132 (16)	133 (15)	17	127.8
20-35	626 (74)	644 (75)	75	116.5
>35	88 (10)	89 (10)	6	67.4
Parity				
0	138 (16)	139 (16)	19	136.7
1	137 (16)	141 (16)	17	120.6
2-4	393(47)	406 (47)	43	105.9
5+	178 (21)	180 (21)	19	105.6
Polygynous household				
Yes	409 (48)	420 (48)	61	145.2
No	437 (52)	446 (52)	37	83.0
Maternal education				
None	678 (80)	694 (80)	78	112.4
Literacy/primary school	116 (14)	120 (14)	17	141.7
Secondary school	52 (06)	52 (06)	3	57.7
Socio-economic status				
(based on household assets)^a^				
Quintile 1 (most poor)	182 (22)	184 (21)	17	92.4
Quintile 2	156 (18)	163 (19)	26	159.5
Quintile 3	178 (21)	178 (21)	21	118.0
Quintile 4	166 (20)	174 (20)	14	80.5
Quintile 5 (least poor)	164 (19)	167 (19)	20	119.8
Previous child death^b^				
Yes	419 (59)	429 (59)	54	125.9
No	289 (41)	298 (41)	25	83.9
Antenatal care visits				
0	237 (28)	240 (28)	25	104.2
1-2	455 (54)	466 (54)	58	124.5
>2	154 (18)	160 (18)	15	93.8
Mother sleeps under bednet				
Yes	324 (38)	332 (38)	32	96.4
No	522 (62)	534 (62)	66	123.6
Birth attendant				
Health personnel	319 (38)	326 (38)	41	125.8
TBA/Family/none	527 (62)	540 (62)	57	105.6
Season of birth				
Dry season (Nov-April)		407 (47)	50	122.9
Rainy (May-Oct)		459 (53)	48	104.6
Newborn gender				
Girl		426 (49)	48	112.7
Boy		440 (51)	50	113.6
Birth weight^c^ (g)				
< 2500		41 (05)	5	122.0
≥ 2500		254 (29)	21	82.7
Missing		571 (66)	72	126.1
Low anthropometric score^d^ at week-3				
Yes		212 (24)	27	127.4
No		511 (59)	21	41.1
Missing		143 (17)	50	349.7
Continuous variables				
				Median (IQR)
Infant z-scores at week-3^d^				
z-score of weight for height				−0.81 (−1.68-0)
z-score of height for age				−0.63 (−1.39-0.17)
z-score of weight for age				−0.90 (−1.61 to (−)0.11)

^**a**^A relative wealth index as a proxy for socio-economic status was based on data collected at recruitment on housing material (walls, floor, windows, roof), and household assets such as possession of the following items: car/truck, motorcycle/scooter, bicycle, mobile phone/telephone, plough and chart. The index was constructed using principal component analysis.

^**b**^Restricted to multiparous mothers.

^**c**^only 295 children had a birth weight recorded.

^**d**^n = 723, includes only those with weight and/or height measured at week-3 (±7 days).

### Infant mortality

A total of 98 infant deaths were recorded, yielding an IMR of 113 per 1000 live births (95% CI: 89–143, Figure 3). Of these, 58 deaths (59%) occurred after 28 days, yielding a post-neonatal infant mortality rate of 67 per 1000 live births (95% CI: 51–88). Overall, neonatal deaths (N = 40) and infant deaths by 6 months of age (N = 75) represented 41% and 76% of all infant deaths, respectively. The distributions of child deaths by maternal and infant baseline characteristics are presented in Table 1. Infant deaths occurred in 23 out of the 24 villages and in 13 of them (54%), IMR exceeded 100 per 1000 live births (Table 2). Two villages, Karfiguéla and Nafona1, had very high IMRs of 286 and 250 per 1000, respectively; both had a health facility (Table 2). However, we found no evidence that the between village variation in observed IMR was higher than might be expected due to chance (LR test, p = 0.32).

**Figure 3:**
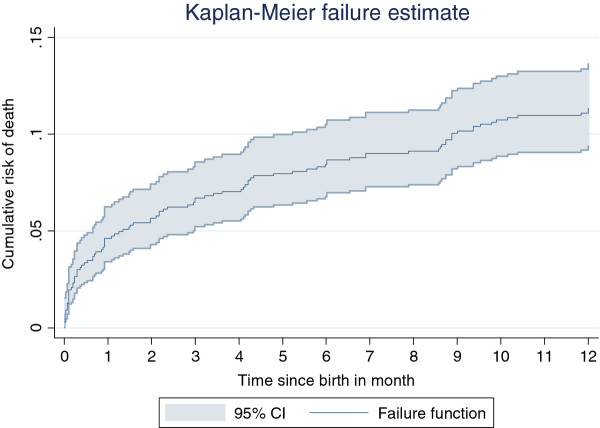
Cumulative risk of infant death with 95% CI in a survival analysis of a cohort of 866 live births in Banfora Health District, Burkina Faso.

**Table 2:** Distribution of facilities, number of live births, infant deaths and infant mortality rates at 6 and 12 months of child age in 24 villages of Banfora Health District (Burkina Faso)

**No**	**Village**	**Health facility exists**	**Distance to health facility (km)**	**Number of live births**	**Twin births**	**Number of infant deaths**	**HIMR**^**a**^	**IMR**^**b**^
							**Per 1000**	**Per 1000**
1	Létiéfesso	No	12	42	0	0	0	0
2	Tangora	No	10	41	2	1	0	24
3	Lémouroudougou cité	No	15	16	0	1	62	62
4	Gouindougouba	Yes	0	44	2	3	45	68
5	Lémouroudougou village	No	10	29	2	2	34	69
6	Kirbina	No	5	28	0	2	71	71
7	Boborola	No	8	41	2	3	49	73
8	Gouin-Gouin	No	5	39	0	3	51	77
9	Kossara	No	4	22	0	2	91	91
10	Tatana	No	3	30	0	3	100	100
11	Kouéré	Yes	0	50	6	5	80	100
12	Niamirandougou	No	8	39	2	4	103	102
13	Tiékouna	Yes	0	28	0	3	71	107
14	Noumousso	No	4	28	2	3	107	107
15	Sikanadjo	No	19	18	0	2	111	111
16	Dêgue-Dêgue	No	14	35	0	4	86	114
17	Damana	No	16	42	0	5	48	119
18	Laferma	No	7	32	2	4	125	125
19	Zédougou	No	11	45	4	6	111	133
20	Tiempangora	No	16	35	0	5	57	143
21	Siniéna	Yes	0	86	6	14	93	163
22	Kotou	No	7	32	0	6	156	187
23	Nafona1	Yes	0	36	8	9	250	250
24	Karfiguéla^**c**^	Yes	0	28	2	8	250	286
Mean (SD)^**d**^		-	-	36(13)	-	4 (3)	90 (61)	112(63)

^**a**^1^st^ half of infancy mortality rate (HIMR, i.e. from 0 to 6 months) is expressed per 1000 live births.

^**b**^Infant mortality rate (IMR) is expressed per 1000 live births.

^**c**^Clusters are ranked by increasing order of IMRs, Nafona1 and Karfiguéla located in Banfora subcounty and both with a health facility, had the highest IMRs in this cohort.

^**d**^Mean (SD) were obtained from cluster-level summaries of the data by village.

Mortality rates (MRs) per 1000 PYOs were very high during the neonatal period (MR = 610, 95% CI: 448–832) and fell rapidly after 4 weeks (MR = 79, 95 CI% CI: 61–102). The average MR from birth to 12 months of age was of 123 (95% CI: 101–150) per 1000 PYOs.

### Probable causes of deaths during the first half of infancy

The distribution of the probable causes of death by 6 months of age (N = 75) is presented in Table 3. During the neonatal period, preterm birth complications (42%) and infections (17%) were the main causes of death while infections (54%) were the major cause of infant deaths in the postneonatal period, dominated by acute respiratory infections (7 cases), malaria (7 cases), meningitis (3 cases) and gastroenteritis (2 cases). The causes of death were unknown for 26 infants (35%), mainly due to missing information from mothers (Table 3).

**Table 3:** Distribution of the probable causes of infant deaths from 0 to 6 months among 75 children in Banfora Health District, Burkina Faso

**Probable causes infant deaths (0–6 months)**	**Neonatal period**	**Postneonatal period**
	**Cases N (%)**	**Cases N (%)**
Infections	7 (17)	19 (54)
▪ Acute respiratory infections (ARI)	3	7
▪ Malaria	0	7
▪ Meningitis	0	3
▪ Gastroenteritis	1	2
▪ Sepsis	2	0
▪ Fever of unknown aetiology	1	0
Preterm birth complications	17 (42)	0
Acute intestinal occlusion syndrome	2 (05)	0
Severe haemorrhages	1 (03)	1 (03)
Birth asphyxia	1 (03)	0
Severe malnutrition	0 (0)	1 (03)
Unknown^**a**^	12 (30)	14 (40)
Total	40	35

^**a**^Either the verbal autopsy could not allow any conclusion from the medical reviewers (5 cases) or the detailed history of death was missing (21 cases).

A total of 67 (89%) deaths by 6 months of age occurred at home, 7 deaths (9%) occurred in a local health facility, and one death occurred in a local healer’s home. Of the children who died before 6 months of age, only 16 (21%) were taken to a health facility prior to death. For 10 of these children (63%), the treatment recorded on the child health card was an antimalarial drug (chloroquine, amodiaquine, or quinine).

### Risk factors for infant death

During the first half of infancy (0–6 months), polygyny, twinship and low anthropometric z-scores at week-3 were factors strongly associated with increased rate of death in univariable Cox regressions (Table 4). Twins had over 8-fold increased rate of death at this age compared to singletons and children with low anthropometric z-score had 4-times higher rate of death compared to their peers with a z-score ≥ −2. There was some weak evidence that a residence closer to health facility, maternal history of child death, being a boy and birth during the dry season were associated with increased rates of infant death (Table 4). After controlling for potential confounders, children from polygynous households, those born to mothers with a previous child death, boys and twin births appeared to experience higher mortality rates between 0 and 6 months of age (Table 5).

**Table 4:** Association of maternal and infant baseline with infant death rate at different child age in univariable Cox regressions in a cohort of 866 live births in Banfora Health District (Burkina Faso)

**Exposure variables**	**0 to 6 months (1**^**st**^**half of infancy)**				**1-12 months (postneonatal period)**				**0 to 12 months (overall infancy)**			
	**PYO**	**No of deaths**	**MRs**^**a**^	**HR**^**b**^	**PYO**	**No of deaths**	**MRs**^**a**^	**HR**^**b**^	**PYO**	**No of deaths**	**MRs**^**a**^	**HR**^**b**^
				**[95% CI]**				**[95% CI]**				**[95% CI]**
Area of residence												
▪ Banfora	198.3	43	217	1.7 [0.8-3.5]	356.3	29	81	1.1 [0.5-2.5]	387.7	56	144	1.6 [0.8-2.9]
▪ Soubakenedougou	102.1	13	127	1	183.9	13	70	1	200.0	18	90	1
▪ Sideradougou	106.6	19	178	1.4 [0.7-2.8]	191.6	16	83	1.2 [0.6-2.4]	208.6	24	115	1.3 [0.7-2.3]
Distance to nearest health facility												
▪ ≤5 km	192.9	44	228	1.5 [0.9-2.7]	346.7	28	81	1.0 [0.6-1.7]	377.5	55	146	1.4 [0.9-2.2]
▪ >5 km	214.	31	145	1	385.2	30	79	1	418.8	43	103	1
Maternal age												
▪ <20	61.9	12	193	1.6 [0.5-5.2]	111.8	9	80	3.1 [0.6-15.0]	121.6	17	140	1.9 [0.6-6.5]
▪ 20-35	302.4	58	192	1	542.6	47	87	1	590.6	75	127	1
▪ >35	42.6	5	11	1.6 [0.6-4.1	77.4	2	26	3.3 [1.0-11.2]	84.1	6	71	1.7 [0.7-4.4]
Maternal parity												
▪ 0	63.8	15	235	1.4 [0.6-3.2]	115.0	10	87	1.0 [0.4-2.3]	125.2	19	152	1.3 [0.6-2.8]
▪ 1	65.9	13	197	1.2 [0.5-2.9]	118.8	11	51	1.1. [0.4 2.9]	129.2	17	131	1.1 [0.5-2.6]
▪ 2-4	193.1	31	160	1.2 [0.6-2.1	346.3	29	58	1	376.9	43	114	1
▪ 5+	84.0	16	190	1	151.8	8	26	0.6 [0.3-1.3]	165.0	19	115	1.0 [0.6-1.7]
History of child death^**c**^												
▪ Yes	201.1	40	199	1.4 [0.8-2.3]	359.6	35	97	1.8 [0.8-4.2]	391.5	54	138	1.5 [1.0-2.3]
▪ No	142.0	20	141	1	257.3	13	50	1	279.5	25	89	1
Polygynous household												
▪ Yes	194.3	47	242	1.8 [1.0-3.3]	345.6	37	107	1.9 [0.9-4.1]	376.7	61	162	1.8 [1.0-3.3]
▪ No	212.6	28	131	1	386.3	21	54	1	419.6	37	88	1
Maternal education												
▪ None	326.8	61	187	1.5 [0.7-3.3]	586.5	49	83	3.8 [0.5-27.0]	638.4	78	122	2.0 [0.9-4.3]
▪ Literacy/primary school	55.2	11	199	1.6 [0.6-4.6]1	99.8	8	80	3.6 [0.4-32.0]	108.5	17	157	2.5 [1.0-6.4]
▪ Secondary school	24.9	3	120	1	45.5	1	22	1	49.4	3	61	1
Socio-economic status^**d**^												
▪ Q1 (most poor)	86.6	13	150	1	157.4	8	51	1	171.1	17	99	1
▪ Q2	73.5	21	286	1.9 [0.8-4.6]	131.2	12	91	1.8 [0.7-4.5]	142.9	26	182	1.8 [0.8-4.1]
▪ Q3	83.8	16	191	1.3 [0.6-2.7]	150.1	13	87	1.7 [0.7-4.2]	163.5	21	128	1.3 [0.7-2.4]
▪ Q4	83.5	11	132	0.9 [0.4-2.1]	151.6	8	53	1.0 [0.3-3.5]	164.5	14	85	0.8 [0.4-1.9]
▪Q5 (less poor)	79.5	14	176	1.2 [0.6-2.3]	141.5	17	120	2.3 [1.1-4.9]	154.2	20	130	1.3 [08–2.0]
Mother sleeps under bednet												
▪ Yes	156.7	25	159	1	284.1	16	56	1	308.7	32	103	1
▪ No	250.2	50	200	1.2 [0.6-2.5]	447.8	42	94	1.6 [1.0-2.8]	487.6	66	135	1.3 [0.7-2.2]
Antenatal care visit												
· 0	112.2	20	178	1.2 [0.5-2.7]	203.1	13	64	0.9 [0.4-1.7]	221.0	25	113	1.1 [0.6-2.0]
· 1-2	218.2	44	201	1.4 [0.7-2.7]	390.5	35	89	1.2 [0.7-2.1]	425.1	58	136	1.3 [0.8-2.2]
· >2	76.5	11	144	1	138.2	10	72	1	150.2	15	100	1
Birth attendant												
▪ Health personnel	154.2	28	181	1	275.4	31	112	1	299.7	41	137	1
▪ TBA/family/other	252.7	47	186	1.0 [0.7-1.5]	456.5	27	59	0.5 [0.3-8]	496.6	57	115	0.8 [0.6-1.2]
Season of delivery												
▪ Dry season (Nov-Apr)	189.1	40	211	1.3 [0.9-2.0]	339.7	26	76	0.9 [0.6-1.3]	369.6	50	135	1.2 [0.8-1.7]
▪ Rainy season (May-Oct)	217.8	35	160	1	392.2	32	81	1	426.7	48	112	1
Sex of newborn												
▪ Girl	201.7	32	158	1	362.8	29	80	1	394.6	48	121	1
▪ Boy	205.2	43	209	1.3 [0.8-2.1]	369.0	29	78	1.0 [0.6-1.6]	401.7	50	124	1.0 [0.7-1.5]
Twin births												
▪ Yes	13.3	18	1349	8.3 [4.8-14.5]	21.5	10	465	6.7 [3.12.7]	24.0	21	876	7.7 [4.6-12.8]
▪ No	393.6	57	145	1	710.4	48	67	1	772.3	77	100	1
Low anthropometric z-scores at week-3^**e**^												
· Yes	101.9	18	177	4.0 [1.5-10.5]	180.3	25	138	3.2 [1.8-5.7]	196.5	27	137	3.2 [1.8-5.9]
· No	253.1	11	43	1	461.0	20	43	1	500.2	21	42	1

^**a**^Mortality rates (MRs) per 1000 person-years of observation (PYO).

^**b**^All crude analyses adjusted for the cluster-design of the main EBF-study and estimates of HRs are with robust standard errors.

^**c**^Restricted only to multiparous mothers.

^**d**^See Table 1 for computation of the index of household assets.

^**e**^Applicable only to those with weight or height measured at week-3 visit (±7 days, N = 723).

**Table 5:** Multivariable analyses of factors associated with infant death rate at different child age in a cohort of 866 live births in Banfora Health District (multivariable Cox regression models)

**Exposures**	**1**^**st**^**half infancy**	**Postneonatal period**	**Overall infancy**
	**HR [95% CI]**	**HR [95% CI]**	**HR [95% CI]**
	** n = 727**	** n = 720**	** n = 727**
	**N = 60 deaths**	**N = 45 deaths**	**N = 79 deaths**
Distance to health facility			
· ≤5 km	1.5 [0.8-3.1]^**a**^		1.6 [0.8-2.8]^**c**^
· >5 km	1		1
History of child death			
· Yes	1.5 [1.0-2.7]^**a**^	1.6 [1.0-2.6]^**c**^	
· No	1	1	
Polygynous household			
· Yes	2.4 [1.3-4.3]^**a**^	2.0 [1.1-3.6]^**b**^	2.4 [1.4-4.0]^**c**^
· No	1	1	1
Mother sleeps under bednet			
· Yes		1	1
· No		1.8 [0.9-3.4]^**b**^	1.3 [0.8-2.1]^**c**^
Season of birth			
· Dry season (Nov-Apr)	1.4 [0.8-2.4]^**a**^		1.4 [0.9-2.1]^**c**^
· Rainy season (May-Oct)	1		1
Sex of newborn			
· Girl	1		1
· Boy	1.8 [1.0-3.1]^**a**^		1.2 [0.8-2.0]^**c**^
Low anthropometric z-scores at week-3 visit			
· Yes		3.3 [1.8-6.0]^**b**^	
· No		1	
Twin births			
· Yes	10.6 [5.4-20.8]^**a**^		8.4 [4.6-15.3]^**c**^
· No	1		1

^**a**^Adjusted for distance to nearest health facility, history of child death, polygyny, season of birth, gender of the newborn, twinship and for clustering.

^**b**^Adjusted for polygyny, maternal use of bednet, anthropometric z-scores at week-3 and for clustering.

^**c**^Adjusted for distance to nearest health facility, history of child death, polygyny, maternal use of bednet, season of birth, gender of the baby, twinship and for clustering.

Adjustment for clustering was done fitting a Cox Gamma shared frailty model in STATA/SE 11.0.

In the postneonatal period, there were 3 variables associated with increased rate of death in univariable analyses (Table 4): infants of mothers not sleeping under bednet (HR = 1.6, 95% CI:1.0-2.8), twin births (HR = 6.7, 95% CI:3.5-12.7) and children with low anthropometric z-scores at week-3 (HR = 3.2, 95% CI:1.8-5.7). There was weak evidence that infants born in polygynous households and those whose mothers had a history of child death were at higher risk of postneonatal death. The adjusted models showed a doubling of the rate of death for children born in polygynous households (HR = 2.0, 95% CI: 1.1-3.6) and over 3-times higher rate of death for children with poor anthropometric status at 3 weeks of age (Table 5). There was a 80% (95% CI: -10 to 240%) higher rate of postneonatal death for infants whose mothers were not sleeping under a bednet.

For the entire period of infancy, there were 3 factors strongly associated with increased rate of infant death (Table 5): maternal history of child death (HR = 1.6, 95% CI: 1.0-2.6), birth in a polygynous household (HR = 2.4, 95% CI: 1.4-4.0) and twin births (HR = 8.4, 95% CI: 4.6-15.3). We found no further evidence of an increased rate of death among children living closer to a health facility in the multivariable models (Table 5). The observed higher rate of death among children living closer to a health facility in the crude analysis (Table 4) was no longer present when the two clusters with the highest IMRs and both with a local facility were excluded (HR = 0.9, 95% CI: 0.6-1.3) or when the cut-off point was set to 10 km (HR = 0.81, 95% CI: 0.5-1.3). We did not find any evidence in multivariable analyses that a birth in the dry season or being a boy was associated with a significantly increased rate of infant death in this cohort (Table 5).

## Discussion

In this community-based prospective cohort study, the IMR was very high. Infections and preterm birth complications were the main probable causes of infant deaths up to 6 months of age. Overall, 2 maternal variables (history of previous child death, polygynous status) and 2 infant characteristics (twin births, poor anthropometric status at 3 weeks of age) were identified as risk factors for infant death.

### Estimates of postneonatal and infant mortality rates

Our estimates of postneonatal mortality rate and IMR are consistent with recent publications on child mortality for Burkina Faso [3,11]. However, these results are almost twice as high as the estimates provided in a recently published provisional report of the 2010-DHS in Burkina Faso, where postneonatal mortality rate and IMR were estimated to 37 and 65 per 1000 live births, respectively [13]. It is important to remember that the estimates provided in DHS are nation-wide averages and could therefore mask within-country variations in the burden of infant deaths. Another limitation of DHS and surveys in general, is the likelihood of recall bias, especially in populations where literacy is very low, such as in Burkina Faso.

We found few recent prospective cohort data on infant mortality in Burkina Faso. In fact, only two community-based studies one conducted in the Nouna’s DSS (North-western part of Burkina) in 2003, and the second in the Oubritenga’s DSS (Central part of the country) in 2000 reported estimates of IMRs lower than ours, at 67.8 and 56.7 per 1000, respectively [21,22]. The context of these two studies may explain the observed difference as several health interventions were going on both sites [21,23-29].

One important reason for the observed high burden of infant death in a rural area of Burkina Faso is the patchy and weak health system, as suggested by other studies [30,31]. Coverage and utilization of health facilities were low in Banfora and concerns about the quality of care have been raised [12,16]. In this study, only a quarter of the villages had a local health facility. Both the proportion of health-facility deliveries and the proportion of children taken to a local health facility prior to death provide evidence of a very low attendance at health centres. Even in the group of children born in a health facility, nearly 10% had no birth weight recorded, and the apparent over-prescription of ineffective drugs (for example chloroquine), are both indicative of the poor quality of care provided in some health centres. It is likely that the overall low attendance at health facilities is in part a reflection of this poor quality of care, either due to poor outcomes among patients, or to little consideration shown to attendants by the health staff.

Other reasons for high IMRs include low maternal education, poverty and understaffing of health facilities, a quasi-permanent triad in the study area. In this study most of mothers had *no* formal education, a quarter was high multiparae and the ratios of health personnel to population were extremely low [12]. Socioeconomic status was measured using household assets but we found no association between the generated wealth index and risk of infant death. In the study area, over 80% of the population has farming as the major source of income and Banfora region is, relatively speaking, one of the less poor areas in this poor country [11,12,32].

Alternative explanations for such high IMR might be selection or reporting bias. However, a random sample of eligible mothers was selected for data collection [15]. We also paid careful attention to the training and field supervisions of data collectors and also to the standardisation of questionnaires and their administration [14,15]. Furthermore, the involvement of “recruiters” from local communities in data collection improved the timeliness of information, as well as the acceptance and confidence granted to our study team. In this prospective cohort study, we made sure data collectors visited mothers and recorded all infant deaths, irrespective of whether the local “recruiter” had notified the case. The VA questionnaire was administered directly to the child’s mother as long as she was available, and interviews were conducted in local languages to reduce misunderstanding. Infant deaths are important social events in the study area and it is very unlikely that a live child would be reported as having died.

### Probable causes of infant deaths by 6 months of age

The most frequent probable causes of deaths identified during the first half of infancy were infections and preterm birth complications, consistent with published literature from Burkina Faso [33-35] and elsewhere [36]. Infections can be worsened by malnutrition [27,37]. The reported high proportions of acute respiratory tract infections and malaria in infant deaths from infectious causes are consistent with the epidemiological patterns of these two diseases in the study area [28,33-35,38]. These findings are also consistent with the observed higher infant death rate in the dry season during the first half of infancy and the lower rate of infant death in the postneonatal period for infants whose mothers were reported to sleep under a bednet. Previous studies in Burkina Faso have reported that mortality due to acute respiratory infections peaks during first half of infancy and during the dry season, and is higher in rural settings [35,38].

### Risk factors for infant death

Of the factors explored in this cohort, polygyny, maternal history of child death, twin births and poor anthropometric status at week-3 were associated with an increased mortality rate.

Polygyny has previously been reported to be associated with increased risk of child death at several ages [39,40] although this is not a consistent finding [41]. We believe this variable could be a surrogate for poor socioeconomic status or an indicator of the women’s status in our settings. Polygyny was common in Banfora and has in some studies, been associated with poor socioeconomic status and parental neglect [11,40].

A maternal history of child death has been reported in several other studies to be associated with an increased risk of infant death including two studies from Burkina [34,42]. Our findings are consistent with those reports. It is likely that persistent harmful practices could contribute.

Twin births were at a very high risk of death in this cohort as found in previous studies in Burkina Faso where twins had over 4-times increased risk of infant death [34,43,44]. Our findings suggest that the death rate remains high for twins until 6 months of age. Multiple births were found in several studies from Africa to have an increased risk of death especially in the neonatal period [16,45,46]. Preterm birth and low birth weight, both frequently associated with multiple births, both increase the risk of early death.

Low anthropometric z-scores at 3 weeks of age were associated with a 3-times higher rate of infant death in the postneonatal period. Because of the number of children who did not have anthropometric data at week-3 and due to the potential interaction between birth weight and history of previous child death (which relates to maternal parity), we did not include this variable in the adjusted models for the period of the first half of infancy or entire infancy. Our data strengthen the existing knowledge on the association between malnutrition and high child mortality [47,48].

A surprising finding at first sight is that of a higher death rate in the group of children living closer to a health facility. This was largely due to the high mortality rates observed in the 2 study clusters with the highest IMRs. Alternative explanations of this result in contrast to previous data from Burkina Faso [22,34], is either the difference in the cut-off point used (as shown) or it may relate to the already mentioned poor quality of care in facilities. The presence of a facility itself will not make any difference if villagers do attend it only in case of complications, if the centre is poorly equipped or if the health staff is not adequately trained.

Our study had some limitations amongst which, its low power to detect risk factors associated with a small increase in infant death rate (example household assets, parity, maternal education and number of ANC visits) and the high proportion of deaths with missing probable causes due both to initial study design and cultural context where detailed description of the circumstance of early child death by the mother was emotionally difficult. Another limitation was the variation in villages’ population that may have resulted in lower probability of women from larger or high fertility villages of being recruited.

Nonetheless, our study is a rarity in Burkina Faso, being a recent prospective, community-based cohort study in a rural area. The use of a random sample of pregnant women for data collection reduced the selection bias to a minimum and we achieved a very high follow-up rate by one year. Another strength of this study was the follow-up of multiple births and their inclusion in the analyses.

## Conclusions

This study showed a very high risk of infant death in a rural area of Burkina Faso. Twin births, poor anthropometric status at week-3, a maternal history of child death and polygyny were the factors associated with an increased risk of infant death. Preterm birth complications and infections were the major causes of death by 6 months. Attendance at health centres at delivery and during infancy illness was low. Further studies are needed to understand the relation of quality of care in health facilities and child survival in rural areas of Burkina Faso. With the observed IMRs in a predominantly rural country, Burkina Faso is unlikely to meet MDG-4 by 2015.

Achieving MDG-4 in this country will require a reduction of infant mortality in rural areas and any efficacious intervention should rely on a comprehensive approach that strengthens the existing health facilities, improves the training of health personnel and treatment of infections and also accounts for the perceived barriers to accessing quality care in health centres.

## Competing interests

The authors declare no conflict of interest.

## Authors’ contributions

AHD, NM, HS and TT have designed the study. AHD conducted the study, performed data analyses and drafted the manuscript. GST contributed to data collection. SC contributed to data analyses. SC, NM, GST, HS and TT contributed to interpretation of the findings. All authors contributed to the writing of, and approved the final manuscript.

## Authors’ information

The PROMISE-EBF study group*

Steering Committee:

Thorkild Tylleskär, Philippe Van de Perre, Eva-Charlotte Ekström, Nicolas

Meda, James K. Tumwine, Chipepo Kankasa, Debra Jackson.

Participating countries and investigators:

Norway*:* Thorkild Tylleskär, Ingunn MS Engebretsen, Lars Thore Fadnes, Eli

Fjeld, Knut Fylkesnes, Jørn Klungsøyr, Anne Nordrehaug-Åstrøm, Øystein

Evjen Olsen, Bjarne Robberstad, Halvor Sommerfelt

France*:* Philippe Van de Perre

Sweden*:* Eva-Charlotte Ekström, Barni Nor

Burkina Faso*:* Nicolas Meda, Abdoulaye Hama Diallo, Thomas W. Ouédraogo, Jeremi Rouamba, Bernadette Traoré, Germain Traoré, Emmanuel Zabsonré

Uganda*:* James K. Tumwine, Caleb Bwengye, Charles Karamagi, Victoria

Nankabirwa, Jolly Nankunda, Grace Ndeezi, Margaret Wandera

Zambia*:* Chipepo Kankasa, Mary Katepa-Bwalya, Chafye Siuluta, Seter Siziya

South Africa: Debra Jackson, Mickey Chopra, Mark Colvin, Tanya Doherty,

Ameena E Goga, Lungiswa Nkonki, David Sanders, Wesley Solomons, Wanga Zembe.

## Funding source

The PROMISE-EBF study was funded by the European Commission Framework Programme-6 under the contract INCO-CT-2004-003660. The sponsor had no responsibility in the design, conduct, analysis, interpretation or publication of the data.

## Pre-publication history

The pre-publication history for this paper can be accessed here:

http://www.biomedcentral.com/1471-2458/12/739/prepub
